# Aerial projection of three-dimensional motion pictures by electro-holography and parabolic mirrors

**DOI:** 10.1038/srep11750

**Published:** 2015-07-08

**Authors:** Takashi Kakue, Takashi Nishitsuji, Tetsuya Kawashima, Keisuke Suzuki, Tomoyoshi Shimobaba, Tomoyoshi Ito

**Affiliations:** 1 Graduate School of Engineering, Chiba University, 1-33 Yayoi-cho, Inage-ku, Chiba 263-8522, Japan

## Abstract

We demonstrate an aerial projection system for reconstructing 3D motion pictures based on holography. The system consists of an optical source, a spatial light modulator corresponding to a display and two parabolic mirrors. The spatial light modulator displays holograms calculated by computer and can reconstruct holographic motion pictures near the surface of the modulator. The two parabolic mirrors can project floating 3D images of the motion pictures formed by the spatial light modulator without mechanical scanning or rotating. In this demonstration, we used a phase-modulation-type spatial light modulator. The number of pixels and the pixel pitch of the modulator were 1,080 × 1,920 and 8.0 μm × 8.0 μm, respectively. The diameter, the height and the focal length of each parabolic mirror were 288 mm, 55 mm and 100 mm, respectively. We succeeded in aerially projecting 3D motion pictures of size ~2.5 mm^3^ by this system constructed by the modulator and mirrors. In addition, by applying a fast computational algorithm for holograms, we achieved hologram calculations at ~12 ms per hologram with 4 CPU cores.

Floating or aerial images are useful and attractive for digital signs, entertainment, amusement, businesses and so forth. There are many types of aerial projection techniques. fVisiOn^1^,^2^ is a glasses-free 3D display which can float 3D images on an empty, flat tabletop. Multiple viewers can observe the floating images from any angle. However, fVisiOn requires more than 100 projectors to display floating images with sufficient motion parallax in the horizontal direction. Consequently, fVisiOn is quite complex and expensive. Conversely, a method which uses a half mirror or an imaging system, such as lenses or mirrors, can also project aerial motion pictures easily. With such a method, a volumetric display^3^ with 3D architecture that can display 3D images is required for floating 3D motion pictures. Volumetric displays consisting of strings^4^,^5^, fog^6^,^7^ and water drops^8^ have been demonstrated. In these cases, projectors are necessary for projecting aerial 3D images, because the volumetric displays only behave as screens. In addition, when a volumetric display comprising a 2D screen such as a diffuser plate is used, scanning or rotating processing is required to display 3D images^9^,^10^,^11^,^12^. For high-definition, clear 3D images with this type of display, mechanical processing at high speed is required and images must be projected onto the display in synchronization with the high-speed mechanical processing. Although light-emitting-type volumetric displays using light-emitting diodes^5^ and quantum dots^13^ can form 3D images without projectors, high definition is impractical because of the amount of electrical wiring or the difficulty of fabrication and construction.

Holography can also project natural 3D images aerially without multiple projectors and mechanical processing^14^,^15^,^16^,^17^,^18^,^19^,^20^,^21^,^22^,^23^,^24^,^25^,^26^,^27^,^28^,^29^,^30^,^31^,^32^,^33^,^34^,^35^,^36^. This technique captures a 2D image, called a hologram, which contains 3D information about objects. We can reconstruct and observe a 3D image of a recorded object with adequate illumination of the hologram. Electro-holography^16^,^18^,^19^,^20^,^23^,^24^,^25^,^26^,^27^,^28^,^29^,^31^,^32^,^33^,^34^,^35^,^36^ enables projection of 3D motion pictures in air because it uses a spatial light modulator (SLM) as a holographic recording material and displays holograms on the SLM. Computer-generated holograms (CGHs), which are holograms generated numerically by computers, are mainly used in electro-holography. By generating CGHs in real time, we can reconstruct 3D motion pictures of not only virtual scenes, such as 3D computer graphics, but also real scenes. However, because the amount of computation required to generate CGHs is huge, it is quite difficult to generate and reconstruct CGHs in real time. Although real-time or video-rate electro-holography has been realized^16^,^18^,^19^,^20^,^23^,^24^,^25^,^28^,^29^,^33^,^36^, it needs high-performance parallel computing systems such as graphics processing unit (GPU) clusters or large-scale field-programmable gate arrays.

To reduce the computational amount, we use image-type CGHs (computer-generated image holograms: CGIHs)^21^. CGIHs can be generated by locating an object near the hologram plane. Because the amount of computation needed for CGHs is generally proportional to the square of distance between the object and the hologram plane, we can considerably reduce the computational amount using CGIHs. CGIHs are capable of real-time reconstruction of 3D images without high-performance parallel computing systems owing to the reduction in computation. However, when we adopt CGIHs, the ability to aerially reconstruct 3D images is lost because CGIHs reconstruct images near the hologram plane. To remedy that problem, we propose a holographic aerial projection system that utilizes two parabolic mirrors to project a floating image of the reconstructed CGIH, and we report experimental results on aerial projection of 3D motion pictures.

## Results

**Figure 1:**
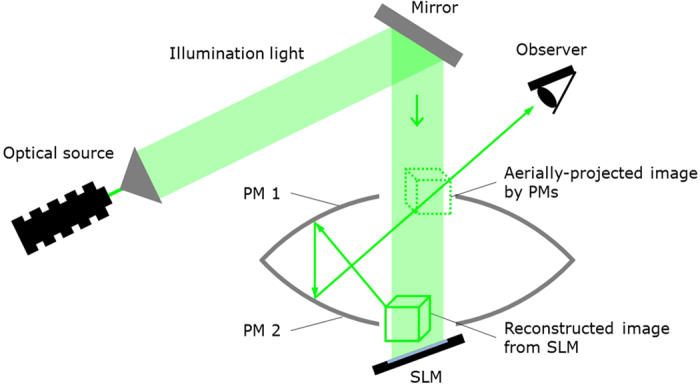
Schematic of the cross-sectional view of the aerial projection system. PM: parabolic mirror; SLM: spatial light modulator.

**Figure 2:**
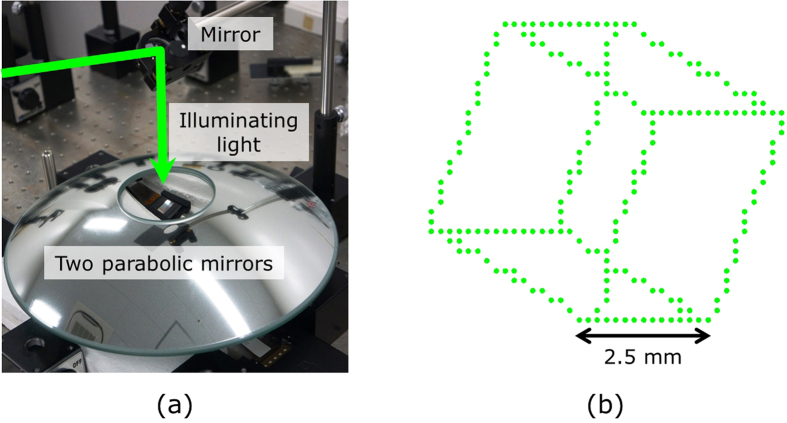
Experimental setup for aerial projection of 3D motion pictures. (**a**) Overhead view. We can see an image of the SLM formed by the parabolic mirrors on the centre of the hole of the upper parabolic mirror. (**b**) Schematic of 3D computer-generated object consisting of 284 point clouds.

**Figure 3:**
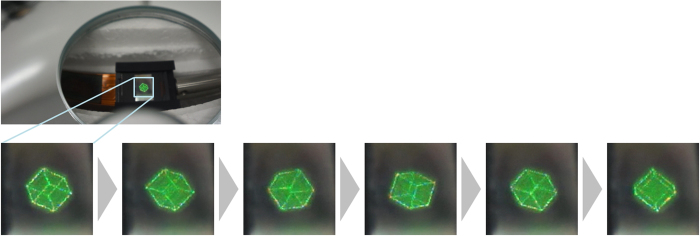
Digital video camera photographs of reconstructed images extracted from a projected 3D motion picture. The image of the SLM formed by the parabolic mirrors can be seen at the centre of the hole of the upper parabolic mirror.

**Figure 4:**
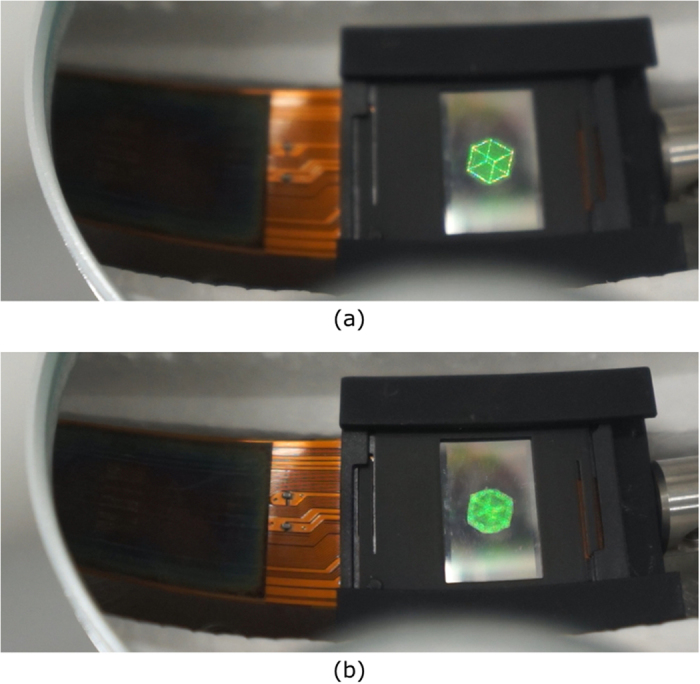
Captured images focusing at (**a**) the rotating cube image and (**b**) the SLM surface.

Figures 1 and 2a show a schematic of the cross-sectional view of the proposed aerial projection system and a photograph of the constructed system, respectively. Two parabolic mirrors made of glass (Shimadzu Rika Co., Japan) are set face to face. The outer diameter, height, inner diameter (hole diameter) and focal length of each parabolic mirror are 288 mm, 55 mm, 80 mm and 100 mm, respectively. To display CGIHs, we use a phase-modulation-type SLM (Holoeye Photonics AG, ‘PLUTO’) and position it ~20 mm away from the bottom of the lower parabolic mirror. The number of pixels and pixel pitch of the SLM are 1,080 × 1,920 pixels and 8.0 μm × 8.0 μm, respectively. Here, the pixel pitch is too coarse to obtain sufficient diffraction angles for hologram reconstruction for large viewing-zone angles; the maximum diffraction angle provided by the SLM is only ~2° in this experiment. With diffraction angles this small, diffraction waves corresponding to reconstructed images of CGIHs are not incident on the mirror surface of the upper parabolic mirror. To solve this issue, we inclined the SLM at ~20° relative to the horizontal plane, as shown in Fig. 1. Diffraction waves from CGIHs are incident on the upper parabolic mirror because of this inclination. A green laser with 532-nm wavelength (Showa Optronics, ‘J150GS’, Japan) is used as an optical source to illuminate the CGIHs. The polarization direction of illuminating light is adjusted by a half-wave plate and a polarizer to obtain the highest diffraction efficiency for the SLM. As an object, a cube consisting of 284 point clouds is used (Fig. 2b). The cube has not only in-plane (or 2D) information but also depth information, and each side of the cube is 2.5 mm. We virtually set the cube ~20 mm away from the hologram plane using the computer and generate CGIHs of a scene in which the cube was rotating. Figure 3 shows six digital video camera images extracted from the projected 3D motion picture (supplementary video). We can see images of a rotating cube clearly and easily. To determine whether or not the images are projected in the air, we varied the focusing distance of the camera. Figure 4a,b show captured images with the focal point at the reconstructed image and at the SLM surface, respectively. In Fig. 4a, the edge of the hole of the upper parabolic mirror is in focus, in addition to the image of the cube. However, in Fig. 4b, the image of the cube is out of focus and blurred while the edge of the SLM is in focus. These images show that the cube’s reconstructed image is aerially projected by the proposed system.

## Discussion

In the results described above, we used CGIHs that were generated before the experiment. However, the proposed system is capable of performing real-time reconstruction, because CGIHs can considerably reduce the computation required for hologram generation. Thus, we can also perform real-time reconstruction of floating 3D motion pictures. We applied Nishitsuji’s algorithm^37^ to calculate CGIHs at high speed. This algorithm uses computer graphics techniques and considers the circular symmetry of zone plates, which are fringe patterns formed by the interference of a spherical wave from a single point cloud and a reference plane wave. To apply the algorithm, we used the following environment: Microsoft Windows 7 Professional Service Pack 1 operating system; Intel Core i7-4790K CPU with 4.0 GHz (we fully used 4 cores) and 8 GB memory; Microsoft Visual C++ 2013 and floating-point computational precision. The computational time is ~12 ms per CGIH. This is sufficient for not only real-time reconstruction but also interactive information presentation to viewers. Here, the size of the CGIHs and the number of point clouds of an object are the same as those described in the Results section.

In this study, we reconstructed only monochromatic 3D motion pictures; however, the system is capable of projecting colour 3D motion pictures by using blue and red lasers in addition to green lasers. Although time-division multiplexing^19^ can be applied to the system, more acceleration of the CGIH calculation would be required, because colour 3D images are formed by three frames—one for each colour.

In the constructed system, the diffraction angle of the SLM is insufficient for holographic reconstruction, so the viewing-zone angle to view the projected motion pictures is restrictively narrow. In addition, the size of the objects (and also the projected images) is too small to recognize whether they contain depth (3D) information. These problems can be solved using an SLM system that has finer pixel pitch and higher resolution, such as 4.8 μm^2^ and 15,360 × 8,640 pixels^29^, respectively. For such a system, we would be able to obtain ~6° of viewing-zone angle and ~40 mm maximum object size.

## Methods

### CGH

**Figure 5:**
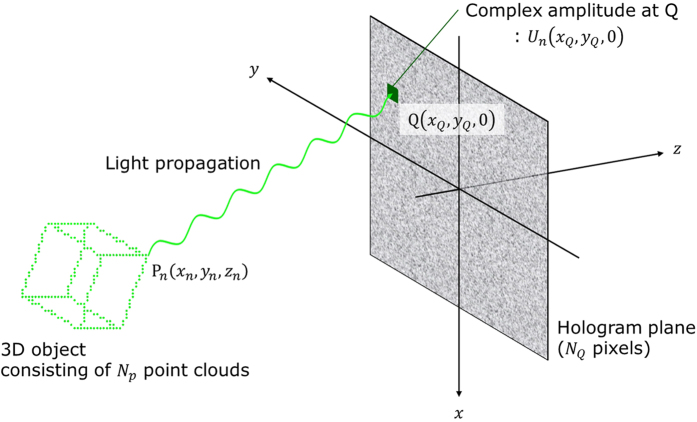
Schematic of the hologram calculation in the recording process of holography.

Figure 5 shows a schematic of the recording process in holography. The hologram plane is on *z* = 0 (or *xy*) plane in Fig. 5. We assume that the object comprises many point clouds, with a particular *n*-th point cloud denoted as P_*n*_(*x*_*n*_, *y*_*n*_, *z*_*n*_) and a pixel on the hologram plane denoted as Q(*x*_*Q*_, *y*_*Q*_, 0). Then *U*_*n*_(*x*_*Q*_, *y*_*Q*_, 0), which means the complex amplitude distribution formed by light emitted from P_*n*_ at Q, is expressed by


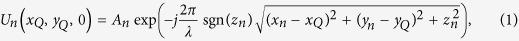


where *A*_*n*_, *λ*, *j* and sgn(*R*) indicate the magnitude of *U*_*n*_(*x*_*Q*_, *y*_*Q*_, 0), the wavelength of light, the imaginary unit and the signum function of a real number *R*, respectively^38^. By using equation (1), the hologram pattern at Q can be calculated by superposing a number of point clouds, *N*_*P*_. There are two types of holograms: amplitude- and phase-modulation types. In this paper, we used kinoform, which is a phase-modulation type hologram. Kinoform *H*(*x*_*Q*_, *y*_*Q*_, 0) is calculated by the following equations:


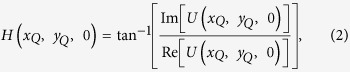



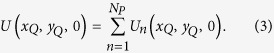


Here, Re[*C*] and Im[*C*] indicate the real and imaginary parts of a complex number *C*, respectively.

Suppose the number of pixels of a CGH is *N*_*Q*_. The amount of computation required to obtain *U*_*n*_(*x*_*Q*_, *y*_*Q*_, 0) is represented by *N*_*P*_ × *N*_*Q*_. In other words, the computational amount is proportional to both *N*_*P*_ and *N*_*Q*_. Therefore, the amount is quite enormous when we generate a high-resolution CGH with a great number of point clouds. However, the range requiring calculation using equation (1) is limited by the pixel pitch *∆p* of the SLM used to display the CGH. Here, *θ*, the diffraction angle of 1st-order diffracted light by a diffraction grating, is given by


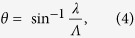


where we assumed that a plane wave is vertically incident on the grating and Λ is the period of the grating^38^. Because Λ corresponds to 2*∆p* in the case of the SLM of the pixel pitch *∆p*, *θ*_max_, the maximum diffraction angle by the SLM, is given by


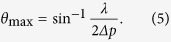


Therefore, because the SLM can only express fringe patterns whose period is larger than 2*∆p*, the radius *r* of the recordable range on the CGH is given by


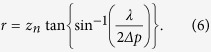


Thus, when the recordable range is a narrow portion of the hologram plane, the computational amount required for a CGH is proportional to the square of *z*_*n*_. We can reduce the computational amount by reducing *z*_*n*_, which is the case for a CGIH.

Aerial projection system using CGIH and parabolic mirrors.

As shown in Fig. 1, the proposed aerial projection system consists of an optical source, an SLM and two parabolic mirrors. A CGIH is displayed on the SLM. When the SLM is illuminated by light emitted from the optical source, a 3D image from the CGIH is reconstructed near the SLM surface. The two parabolic mirrors PM1 and PM2 are set face to face, as shown in Fig. 1. The shapes and focal lengths of PM1 and PM2 are the same, and PM1 and PM2 have a hole at the top and bottom, respectively. When the focal point of PM1 is at the centre of the PM2’s hole and vice versa, an object’s floating image located within PM2’s hole is projected on PM1’s hole. By setting the SLM near PM2’s hole, the reconstructed image from the CGIH corresponds to the object, and the floating image of the reconstructed image is projected on PM1’s hole. This system also enables projection of 3D motion pictures aerially by switching CGIHs. Note that any type of holograms is achievable with the appropriate SLM. In addition, note that a transmissive SLM is also possible, though we show a reflective SLM in Fig. 1.

## Additional Information

**How to cite this article**: Kakue, T. *et al.* Aerial projection of three-dimensional motion pictures by electro-holography and parabolic mirrors. *Sci. Rep.* **5**, 11750; doi: 10.1038/srep11750 (2015).

## Supplementary Material

Supplementary Video

Supplementary Information
