# Supplementary material for: Aerial projection of three-dimensional motion pictures by electro-holography and parabolic mirrors
Source: Sci Rep. 2015 Jul 8;5:11750. doi: 10.1038/srep11750 (PMC4648394; doi:10.1038/srep11750)
Supplement: Supplementary Information [file srep11750-s2.pdf]

Supplementary Information: Aerial projection of three-dimensional motion pictures by electro-holography and parabolic mirrors

Takashi Kakue*, Takashi Nishitsuji, Tetsuya Kawashima, Keisuke Suzuki, Tomoyoshi Shimobaba and Tomoyoshi Ito

Graduate School of Engineering, Chiba University, 1-33 Yayoi-cho, Inage-ku, Chiba 263-8522, Japan

*t-kakue@chiba-u.jp

Video Legend: The supplementary video shows the projected 3D motion picture. We can observe a rotating cube clearly and easily.
